# Modulation of the cognitive impairment associated with Alzheimer’s disease by valproic acid: possible drug repurposing

**DOI:** 10.1007/s10787-025-01695-0

**Published:** 2025-03-19

**Authors:** Mirna Ezzat Sedrak Sorial, Ragwa Mansour Abdelghany, Nesrine Salah El Dine El Sayed

**Affiliations:** 1 Pharmacology and Toxicology Department, Faculty of Pharmacy and Biotechnology, German University in Cairo-GUC, Cairo, Egypt; 2 Pharmacolgy and Toxicology Department, Faculty of Pharmacy, Cairo University, Giza, Egypt

**Keywords:** Alzheimer’s disease, Intracerebroventricular streptozotocin, Valproic acid, Type 3 diabetes, Insulin signaling

## Abstract

Sporadic Alzheimer’s disease is a progressive neurodegenerative disorder affecting the central nervous system. Its main two hallmarks are extracellular deposition of aggregated amyloid beta resulting in senile plaques and intracellular hyperphosphorylated tau proteins forming neuro-fibrillary tangles. As those processes are promoted by the glycogen synthase kinase-3 enzyme, GSK3 inhibitors may be of therapeutic value in SAD. GSK3 is also inhibited by the action of insulin on insulin signaling. Insulin receptor desensitization in the brain is hypothesized to cause inhibition of insulin signaling pathway that ultimately causes cognitive deficits seen in SAD. In extant research, induction of cognitive impairment is achieved by intracerebroventricular injection of streptozotocin—a diabetogenic compound that causes desensitization to insulin receptors in the brain leading to the appearance of most of the SAD signs and symptoms. Valproic acid —a histone deacetylase inhibitor and anti-epileptic drug—has been recently studied in the management of SAD as a possible GSK3 inhibitor. Accordingly, the aim of the present study is to explore the role of multiple VPA doses on the downstream effects of the insulin signaling pathway in ICV STZ-injected mice and suggest a possible mechanism of VPA action. ICV STZ-injected mice showed deficiency in short- and long-term memory as well as increased anxiety, as established by open field test, Modified Y-maze, Morris water maze, and elevated plus maze neurobehavioral tests.

## Introduction

Sporadic Alzheimer’s disease (SAD) is the most prevalent type of dementia affecting the elderly. It is a multifactorial disorder, since several pathways are involved in its pathophysiology, including the insulin signaling pathway, ultimately ending in neuronal death and cognitive dysfunction (Burillo et al. 2021; Colom-Cadena et al. 2020). The major neuropathological hallmarks of SAD are neuro-fibrillary tangles (NFTs) comprised of hyperphosphorylated tau protein (p-Tau) and aggregated amyloid beta (Aβ), leading to the senile plaque formation (Khallaf et al. 2017; Yang et al. 2023).

The predominant regulatory pathway for glycogen synthase kinase 3 (GSK3)—with its two isoforms alpha and beta—is the insulin signaling (Ramasubbu and Devi Rajeswari 2022). Stimulation of the insulin receptor activates this signaling pathway by insulin receptor substance (IRS) and subsequent Akt activation. Active Akt causes the phosphorylation of GSK3 alpha at Serine 21 and GSK3 beta at Serine 9, inhibiting their activity. In SAD patients’ brain, along with insulin resistance, the active form of GSK3 has been previously found to be significantly increased, highlighting the role of GSK3 in this degenerative condition (Leroy et al. 2007; Santos Alves et al. 2023).

GSK3 is the most efficient Tau kinase, leading to its hyperphosphorylation and aggregation into fibrils. It phosphorylates Tau at several sites—including Serine 396 and Serine 404, which impairs the Tau’s ability to promote microtubule assembly. Furthermore, GSK3 plays a role in amyloid precursor protein (APP) processing, thus increasing Aβ formation. In extant studies, GSK3 was also found to facilitate Aβ-induced toxicity. Active GSK3 is the primary kinase that phosphorylates beta-catenin, ultimately resulting in its downregulation and apoptotic cell death in SAD (Lauretti et al. 2020).

Streptozotocin (STZ) is a beta-cytotoxic substance due to the presence of a glucose moiety in its structure. Numerous behavioral, neurochemical and structural features that resemble those found in human SAD have been observed in animals injected with STZ. Thus, SAD is currently considered as Type 3 diabetes (Maciej Dobosz, Gracjan Rudziński, Zuzanna Chilimoniuk and Piotr Więsyk, Natalia Kusak 2023; Salkovic-Petrisic and Hoyer 2007).

Histone deacetylases (HDACs) have been shown to play an inhibitory role in the insulin signaling pathway. Current data suggest a connection between GSK3 and HDACs, given that HDAC3 was demonstrated to be directly phosphorylated by GSK3, inducing neuron death. Conversely, GSK3 inhibition served as a protection against neurotoxicity induced by HDAC3. Therefore, considerable research effort has been dedicated to finding novel GSK3/HDAC dual inhibitors for SAD management (De Simone et al. 2019; Kilgore et al. 2010).

To contribute to this endeavor, as a part of the current study, the role of Valproic acid (VPA)—as one of HDAC inhibitors also known to have an inhibitory effect on the GSK3 enzyme—in managing STZ animal model of SAD was investigated by examining its downstream effects on IR signaling. The required cognitive function data was obtained through neurobehavioral (NB) studies along with the measurement of different molecular components as a part of ex vivo investigations. The effect of different VPA doses was also evaluated in comparison to lithium chloride (LiCl)—an established GSK3 inhibitor—which plays a role in the IR signaling and was thus used as the positive control in this study.

## Methods

### Experimental animals

Adult male Swiss Albino mice weighing from 20 to 25 g, aged between 3 and 4 months, served as study subjects. The animals were obtained from the animal colony of National Institute of Research (Giza, Egypt). Mice were housed in a temperature-controlled room (23 − 34 °C) under a 12-h light/dark cycle, with free access to food and water. Animal procedures were performed after the approval of the Ethics Committee of the German University in Cairo (GUC, project ID PB-2016–02 RMA) in association with the recommendations of the National Institutes of Health Guide for Care and Use of Laboratory Animals (Publication No. 85–23, revised 1985). All efforts were made to minimize animal discomfort and suffering.

### Induction of SAD

The freehand intracerebroventricular (ICV) procedure described by Pelleymounter and colleagues (Pelleymounter et al. 2000, 2002) and subsequently modified by Warnock (Warnock 2007), was adapted for the present investigation to avoid the possibility of penetrating the cerebral vein running along the midline.

### Drugs and treatment schedule

STZ and sodium valproate (Sigma-Aldrich, Germany) and LiCl (Middle East Chemical, MEC, Egypt) were dissolved in 0.9% saline. The mice were divided into eight groups, each with 12 animals. Two control groups were also established, receiving 0.9% saline for three weeks via IP and ICV, respectively. As no statistically significant differences were noted between the results obtained for the two groups, they were combined into one group denoted as Normal. Multiple doses of VPA were used in treatment and the largest dose—200 mg/kg—was given alone to one group in order to check if the drug caused any detrimental effect to the animals. Accordingly, Group 1 comprised mice from the Normal group (receiving either IP or ICV saline for 3 weeks), while mice in Group 2 were injected with STZ (one ICV injection, 3 mg/kg) and they represented the diseased group (Deshmukh et al. 2009; Shi et al., 2017). Group 3 was directly injected with 200 mg of VPA alone for 21 days. Group 4 represented the positive reference, receiving STZ (one ICV injection, 3 mg/kg), followed by LiCl (120 mg/kg, IP) administration five hours later, for three weeks (Sharma and Taliyan 2015). Mice in Groups 5, 6, 7 and 8 were injected with STZ (one ICV injection, 3 mg/kg), followed by VPA (50, 100, 150 and 200 mg/kg, IP) given five hours later, for three weeks (Kilgore et al. 2010; Nalivaeva et al. 2012; Xuan et al. 2015). NB tests were performed within 24 h after the last day of injection.

### Behavioral assessment

#### Open field test (OFT)

OFT is an extensively used test to measure changes in locomotor and exploratory behavior associated with psychological, genetic, experiential, physiological and pharmacological manipulations. The test apparatus consists of a square-bottomed box with an open ceiling and a floor that is divided into 16 equal squares. During the habituation day, each mouse was positioned in the central area and was left to get acquainted to the field for three minutes before being removed. On the test day, each mouse was again left to explore the field for three minutes, during which ambulation, rearing and grooming frequencies were recorded (Ahmed et al. 2011).

#### Modified Y-maze (MYM)

MYM is used for assessing the short-term and long-term spatial recognition memory in animals, capitalizing on their innate inclination to explore novel environments. The MYM apparatus consists of three wooden arms forming a Y shape. The test performed as a part of this study consisted of a 5-min sample trial (T1) followed by a 5-min retrieval trial (T2) separated by inter-trial period that was either 30-min long to assess short-term memory (STM) or 24-h long to assess long-term memory (LTM). In T1, each mouse was allowed to explore two arms of the maze, while the third arm was blocked. In T2, the block was removed and each mouse was allowed to explore all three arms. The previously blocked arm was defined as the novel arm. During T2, the time spent in each arm as well as the number of entries into each arm were recorded. Percentage of time spent in and the percentage of visits to the novel arm were calculated (Martini et al. 2014; Sanderson et al. 2009; Wolf et al. 2016).

#### Morris Water Maze (MWM)

MWM test is one of the most established methods in the assessment of spatial memory deficits arising from hippocampal defects. The apparatus comprises a stainless-steel pool filled with water and featuring an underwater platform. For the present study, visibility in the water was abrogated using a purple non-toxic dye to hide the platform, ensuring that no visual aids were present to affect animal behavior. The pool was randomly sectioned into four equal quadrants using thin ropes tied to its edges to form an X-shape. The quadrant in which the platform was located was denoted as the target quadrant. In the initial 4-day phase, each mouse was given two consecutive training trials per day, with a gap period of at least 15 min. The animal was gently lowered into the water to face the wall of the pool. Each trial lasted no more than 120 s, beyond which if the mouse had failed to reach the platform, it was gently aided onto the platform, and was left there for 20 s before removal. Mice that successfully found the platform within the initial 120 s were also given the additional 20 s on the platform before removal. The time taken by each animal to reach the platform, known as the mean escape latency (MEL) time was recorded for each animal for each of the eight trials, averaging each of the two trials performed on the same day. The MEL for the 4th day was used as an acquisition/learning index. On the 5th day, the platform was removed, and a probe test was carried out, whereby each animal was permitted to explore the pool for 60 s. The time spent by each mouse in the target quadrant was used as a retrieval/memory index (De Coninck et al. 2017; Gupta and Gupta 2012; Sodhi and Singh 2013).

#### Elevated Plus Maze (EPM)

EPM was used to test for both memory acquisition and retention, as well as anxiety. EPM apparatus used for this purpose consisted of a white wooden box with four arms set perpendicular to the central area. Two of the arms were closed by vertical walls while the other two were left open. The maze was raised about 40 cm from the ground. For half of the mice in each group, each animal was positioned at the end of either of the open arms on the first day and the number of seconds it took to reach one of the closed arms was recorded as initial transfer latency (ITL). If a mouse failed to find the closed arm within 90 s, it was gently guided to one of the closed arms. Irrespective of the success rate, each mouse was permitted to explore the maze for 20 s before being returned to its cage. On the second day, retention of this learned task was examined to assess memory, whereby a significant reduction in the retention transfer latency (RTL) value indicated memory improvement. For the other half of each group, each mouse was placed in the central area and was left to explore the maze for five minutes and the time spent in both open arms was recorded. In addition, the number of entry attempts was recorded for both open and closed arms. Results were expressed as time spent in open arms and percent of open arm entries. In line with previous studies, more time was spent in the closed arms by animals with high anxiety levels (Okonogi et al. 2018; Parle and Bansal 2011).

### Tissue sampling

Following NB tests, mice were sacrificed by cervical dislocation and decapitation. The site of ICV injection was assessed by visual inspection of the dissected brain, in accordance with the standard procedure commonly implemented to confirm the technique accuracy. Results pertaining to mice presenting with misplacement of the injection site or any sign of cerebral hemorrhage were excluded from the statistical analyses. However, this occurred in less than 5% of the sample. Next, the brains were divided into two hemispheres, weighed and homogenized as a 10% homogenate in a proper volume of an appropriate buffer for each technique. Homogenization was performed using Potter homogenizer, at a speed 3 (14,000 rpm) for 20 s. The supernatants were kept at -80 C^o^ until required for the ex vivo assay. The rest of the kits were stored in a no-frost freezer at -20 ^0^C.

### Western Blotting (WB)

The WB technique was adopted to determine the p-IRS-1, p-GSK3 alpha, p-GSK3 beta, p-Tau and p-Beta catenin levels. The specific steps implemented in our research was as follows: 1 ml ice cold RIPA buffer was added to tissue sample and sonicated for 30 s at 1 min intervals. Lysates were centrifuged at 16,000 $\times$ g for 30 min at 4 °C. Supernatants were then transferred to fresh cold tubes and protein concentration was measured using a Bradford Protein Assay kit (SK3041, BIO BASIC, canada). 5 – 20 µg of protein were added to an equivalent volume of 2 $\times$ Laemmli sample buffer, the pH was adjusted to 6.8, and denaturation of the samples occurred at 95 °C for 5 min using a thermomixer. Samples were separated using SDS-PAGE TGX Stain-Free FastCast (SDS-PAGE 161–0181, Bio-Rad Laboratories, USA). 20 µg of total protein was loaded in each well. Proteins were transferred onto PVDF membrane then blocked with 3% BSA in tris-buffered saline with 0.1% Tween 20 (TBST) buffer for 1 h at room temperature. Primary antibodies against p-IRS 1 (44-816G), p-GSK3 alpha (MA5-15,021), p-GSK3 beta (MA5-14,873), p-Tau (44-752G), p-Beta catenin (PA5-37,543) and beta actin (PA1-46,296, Thermofisher, USA) were diluted in TBST to a final concentration of 1:1000. Primary antibody solutions were incubated with the membranes overnight at 4 °C, after which membranes were washed 3 – 5 times in TBST. HRP-conjugated secondary antibody (32,460), used at 1:5000, were incubated with the membranes for 1 h at room temperature, followed by 3 – 5 washing steps. Then, chemiluminescent substrate was added to the membrane for 5 min at room temperature in the dark and then visualized using a CCD camera based ChemiDoc MP^™^ imager. ImageJ (NIH) image analysis software was used to quantify band intensities. All samples were normalized against their respective beta-actin bands.

### Estimating beta amyloid protein (βAP) levels

βAP levels were assayed using βAP ELISA kit (MBS733133, MyBiosource, USA). This assay employs the quantitative sandwich enzyme immunoassay technique (Engvall and Perlmann 1971). In short, A polyclonal antibody specific for βAP was pre-coated onto a microplate. Standards and samples were pipetted into the wells and any βAP present was bound to the immobilized antibody. After washing, HRP-conjugated polyclonal antibody specific for βAP is added to each well to bind to the βAP immobilized on the plate, for its quantitative determination. After washing away any unbound enzyme, a substrate solution was added, whereby color developed in proportion to the amount of βAP bound in the initial step. The color development was terminated and the color intensity was measured and compared to a standardized curve.

### Estimating levels of HDAC3 gene expression using qPCR technique

Total RNA was isolated using Qiagen tissue extraction kit (USA) and the calculations similar to similar to those employed by Livack and Schmittgen (2001) (Livak and Schmittgen 2001). Reverse transcription process was performed using two µg RNA sample using high capacity cDNA reverse transcription kit, thermo-fischer (Applied Biosystems). MultiScribe™ reverse transcriptase was used for synthesis of cDNA from RNA. RNase inhibitor was used for inhibition of RNase activity. qPCR amplification and analysis were done with the aid of an Applied Biosystems version 3.1 (StepOne^™^, USA). The qPCR test with the primer sets (Invitrogen, USA) were optimized at the annealing temperature. The amplified sequence was visualized using SYBR green dye (Applied Biosystems). For each sample, reactions were set in duplicates and the expression levels of target genes were normalized to the GAPDH levels, which served as an internal standard control. Relative expression was calculated with respect to the control group, whereby gene expression levels were determined using the 2^−∆∆Ct^ method.

### Statistical analysis

Statistical analyses were performed using Graph Pad Prism 8 software and the obtained results were represented as Mean ± SEM. The results yielded by the NB tests were analyzed through the use of one-way analysis of variance (ANOVA) followed by Tukey − Kramer post-hoc test, with a p value < 0.05 considered statistically significant. The confidence interval was fixed at 95%.

## Results

### OFT results

No statistically significant differences were noted between any of the groups with respect to the OFT results, indicating normal locomotor behavior in mice in all groups (supplementary Fig. 1).

### MYM results

In both STM and LTM tests, the STZ group showed a significant decrease in both % of time spent in novel arm and % of novel arm entries when compared to the Normal group. Treatment with LiCl and VPA increased both parameters significantly when compared to the STZ group (supplementary Figs. 2 − 5).

### MWM results

On the 1st day, all groups achieved the same initial MEL value. The MEL of the STZ group remained statistically significantly higher on day 2, 3 and 4 when compared to the Normal group. The LiCl group showed significant improvement starting from the 2nd day. All VPA-treated groups showed continuous improvement as their MEL was significantly lower than in the STZ group on the 2nd, 3rd and 4th day. The STZ group spent significantly less time in the target quadrant than the Normal group. Treatment with LiCl increased mean time spent in the target quadrant compared to the STZ group; however, the increase was not statistically significant. On the other hand, a statistically significant increase in mean time spent in the target quadrant was noted for the groups receiving VPA compared to the STZ group (supplementary Figs. 6 and 7).

### EPM results

#### Effect on memory

A statistically significant difference between ITL and RTL was noted for the first half of the animals in all groups (except STZ). Moreover, the STZ group had a higher RTL relative to the LiCL and VPA-treated groups, as well as the Normal group, but only in the latter case this difference was statistically significant. (Supplementary Figs. 8 and 9).

#### Effect on anxiety levels

For the other half of animals, the STZ group had significantly lower results in both the time spent in the open arms and the % of arm entries in comparison to the remaining mouse groups (supplementary Figs. 10 and 11).

### Estimation of p-IRS-1 protein levels using the WB technique

**Fig. 1:**
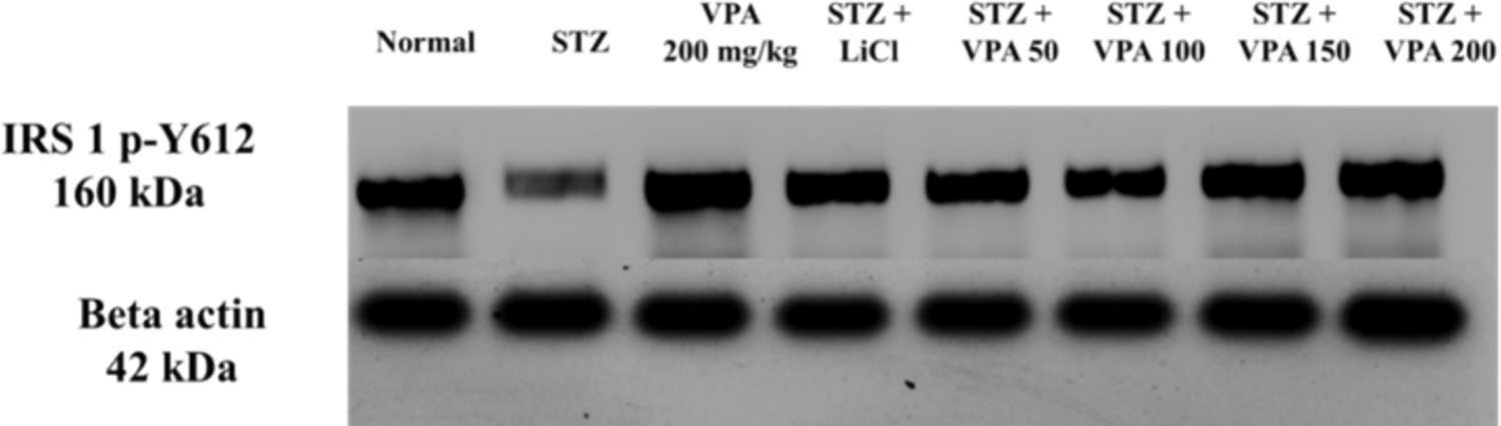
p-IRS-1 results

The p-IRS-1 protein levels in the STZ group were significantly lower compared to the Normal group, as well as to groups treated with LiCl and VPA, as shown in Fig. 1 and supplementary Fig. 12.

#### Estimation of p-GSK3 alpha protein levels using the WB technique

**Fig. 2:**
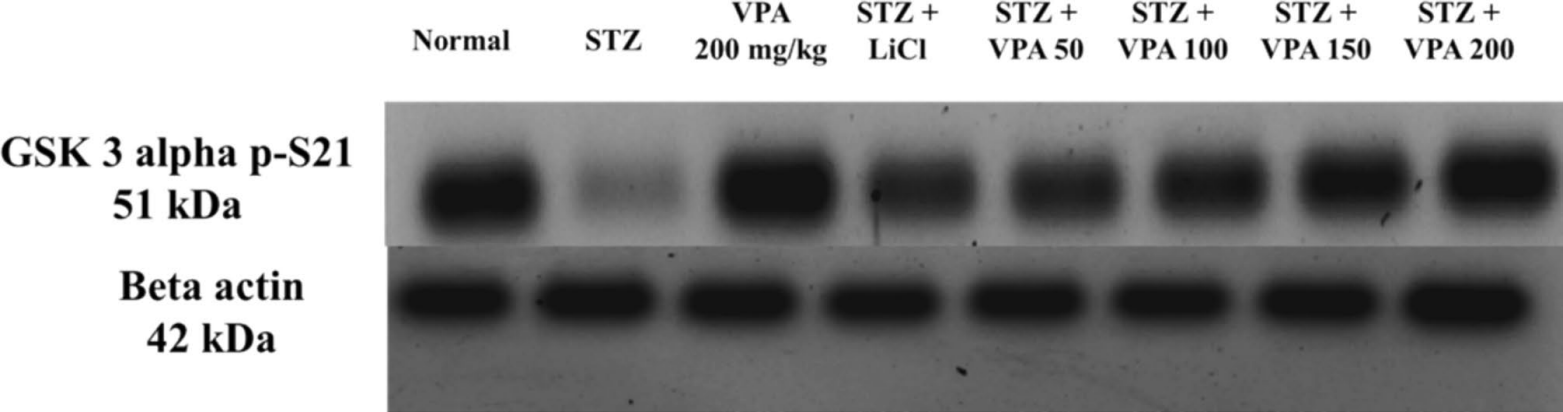
p-GSK3 alpha results

The p-GSK3 alpha protein levels in the STZ group were significantly lower compared to not only the Normal group but also the LiCl and VPA-treated groups, as shown in Fig. 2 and supplementary Fig. 13.

### Estimation of p-GSK3 beta protein levels using the WB technique

**Fig. 3:**
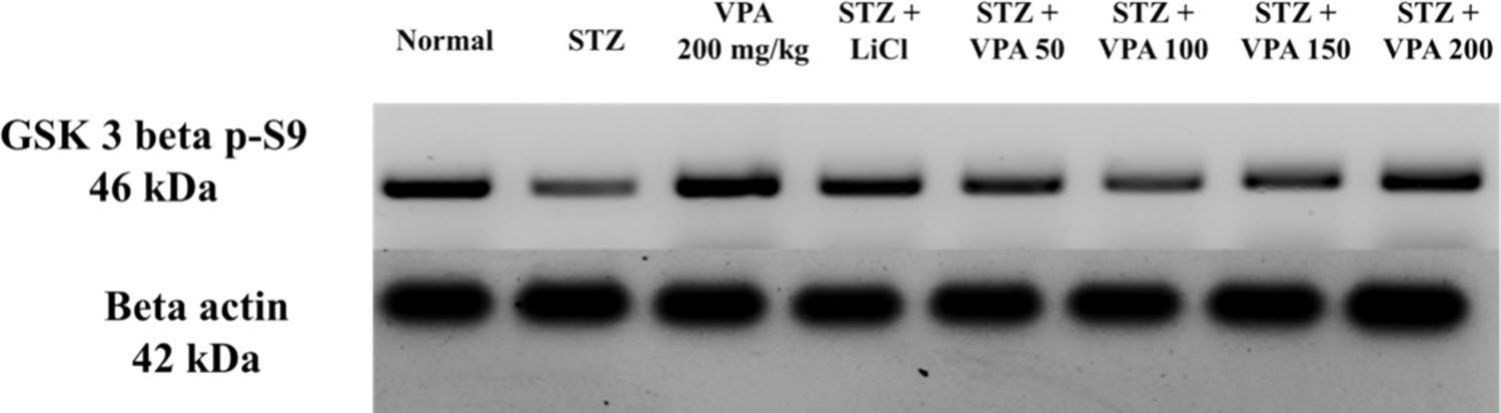
p-GSK3 beta results

The p-GSK3 beta protein levels were also significantly lower in the STZ group compared to the Normal group, as well as the LiCl- and VPA-treated groups, as shown in Fig. 3 and supplementary Fig. 14.

### Estimation of p-Tau protein levels using the WB technique

**Fig. 4:**
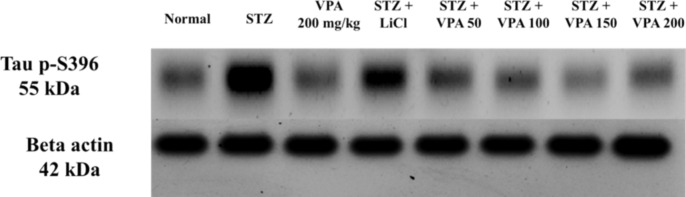
p-Tau results

The STZ group showed a statistically significant increase in the p-Tau protein values when compared to the Normal group. While a significant decrease in the relative p-Tau protein levels was noted for the LiCl group compared to the STZ group, the values were still significantly increased when compared to the Normal group, as well as those treated with VPA 100 mg/kg, VPA 150 mg/kg and VPA 200 mg/kg. All VPA-treated groups showed significant decrease in the relative p-Tau protein levels compared to the STZ group. The p-Tau levels for groups treated with VPA at 50 mg/kg and 100 mg/kg were significantly higher than in the Normal group, and a statistically significant difference was also noted between the VPA 50 mg/kg group and VPA 150 mg/kg and VPA 200 mg/kg with respect to the p-Tau values, as shown in Fig. 4 and supplementary Fig. 15.

### .

#### Estimation of p-Beta catenin protein levels using the WB technique

**Fig. 5:**
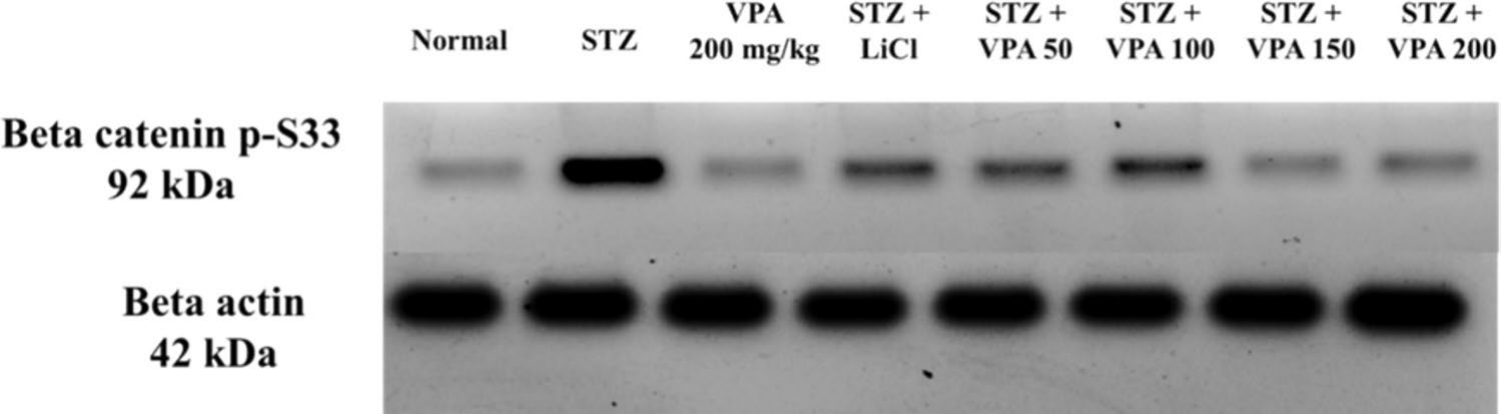
p-Beta catenin results

The STZ group showed a significant increase in the p-Beta catenin protein levels compared to the Normal group, whereas these values were significantly lower in the LiCl group relative to the STZ group but remained significantly higher than in the Normal group. All VPA-treated groups showed a significant decrease in the relative p-Beta catenin protein levels compared to the ICV STZ group, as shown in Fig. 5 and supplementary Fig. 16.

### Estimation of βAP protein levels using ELISA kit

The STZ group had a statistically higher βAP concentration when compared to the Normal group. On the other hand, its value was significantly lower in the LiCl group as well as the VPA-treated groups (supplementary Fig. 17).

#### Estimation of HDAC3 gene expression levels using qPCR technique

The STZ group showed a significant increase in the HDAC3 expression level relative to the Normal group. In the LiCl group, its value was significantly lower than in the STZ group, but was nonetheless significantly higher than the concentration measured for the Normal group. It was also statistically significantly higher than in the VPA 150 mg/kg and 200 mg/kg groups. While all VPA-treated groups showed a significant decrease in HDAC3 expression compared to the STZ group, a statistically significant difference was also noted for the VPA 200 mg/kg relative to the VPA 50 mg/kg and VPA 100 mg/kg groups (supplementary Fig. 18).

## Discussion

The importance of insulin signaling in the brain stems from its significant role in several functions such as eating, learning, behaviors and cognitive functions. Moreover, it protects the brain from neuroinflammation and redox stress. Brain insulin resistance is found to affect not only neuronal glucose metabolism but also Aβ and Tau pathological evolution, contributing to the SAD pathogenesis (Burillo et al. 2021). As STZ is particularly toxic to insulin-producing cells, it has been extensively used as an experimental model for peripheral toxicity as well as neurotoxicity by inducing insulin resistance (Akhtar et al. 2021). In the study conducted by Hoyer (2002), ICV STZ injection induced similar molecular changes as those noted in SAD patients (Hoyer 2002).

Most notably, ICV STZ has been revealed to play a crucial role in memory and cognitive impairment in the MYM, MWM and EPM tests, which are used for the assessment of STM, LTM, spatial memory and recognition memory (Agrawal et al. 2009; Bokare et al. 2018; Dhull et al. 2012; El Halawany et al. 2017; Ghoneum and El Sayed 2021; Khalili & Hamzeh 2010; Mehan et al. 2011; Noble et al. 2003; A. Singh & Kumar 2016; Weerateerangkull et al. 2008). As demonstrated in the present study, VPA caused an enhancement in memory retention and consolidation, as indicated by the results of the abovementioned tests, which are in line with previous findings on several AD animal models (Qing et al. 2008; Sorial & El Sayed 2017; Wu et al. 2013; Yao et al. 2014).

In addition to memory tests, anxiety-like behavior was assessed using the EPM test and the results revealed that STZ increased anxiety by decreasing the number of open arm entries while VPA had an anxiolytic effect, as indicated by the increased entries to the open arm in the EPM test relative to the STZ group. In several studies, both transgenic mice and STZ-injected mice were found to spend less time in the open arm and make fewer open arm entries than the controls (Chen et al. 2013, 2014; Pinton et al. 2011). Furthermore, in their study focusing on post-traumatic stress disorder, PTSD, Wilson et al. (2014) noted greater open arm occupancy by the VPA-treated group when compared to the untreated group (Wilson et al. 2014).

In the present study, glucose uptake was markedly reduced by brain cells after ICV STZ injection. In prior studies, IR desensitization through a reduced active phosphorylated form was noted (X. Wang et al. 2010), resulting in the inhibition of the receptor activity and downregulation of the insulin signaling pathway (Yamini et al. 2022). ICV STZ was also previously found to inhibit the IRS-1 activation in rats (Song et al. 2014). Similarly, in aged transgenic mice associated with AD, IRS-1 phosphorylation at tyrosine 608 was found to be reduced when compared to control mice (Ong et al. 2015)**.**

Extant research further shows that Akt expression and activity is significantly reduced in ICV STZ-injected animal brains, leading to GSK3 inhibition as well as Aβ accumulation (Chen et al. 2012; Lester-Coll et al. 2006; Salkovic-Petrisic et al. 2006) and Tau hyperphosphorylation at several sites including Ser396, Thr181 and Ser404 (Grünblatt et al. 2007; Shi et al. 2017; Zhou et al. 2013). Although tau acetylation is currently a very interesting point for research in SAD as it has been found to contribute to memory deficits and disrupted mitochondrial assembly (Bryan et al. 2024; Liu et al. 2023), our focus was on phosphorylated tau as it was directly affected by insulin resistance and GSK 3 activation caused by ICV STZ injection.

GSK3 enzyme is essential in the downstream IR signaling that was recently found to be disrupted in both human and SAD animal models. Specifically, Shi et al. (2020) established that increased GSK3β activity in mice caused a reduction in learning with aggregation of Aβ and hyperphosphorylation of Tau (Xiao-Long Shi †, Ning Yan †, 2020)(Hooper et al. 2007)GSK3 was also found by Sirvani et al. (2021) to inactivate IRS due to which all downstream insulin signaling was affected, contributing to insulin desensitization of ICV STZ (Srivani et al. 2021). Phosphorylation of β-catenin by GSK3β at Ser33, Ser37 and Thr41 were found by Molagoda et al. (2022) to lead to its degradation, while inhibition of GSK3β lead to β-catenin activation (Molagoda et al. 2021). In the study conducted by Ghanevati and Miller (2005), the p-β-catenin level was increased dramatically in the brains of SAD patients when compared to a normal control (Ghanevati and Miller 2005).

Coinciding with our results, in the study conducted by Zhang et al. (2017), the expression of HDAC3 mRNA and protein levels were significantly augmented in SAD transgenic mice compared with wild type mice (J. Zhang et al. 2017). According to Zhu et al. (2017), a significant increase in HDAC3 expression in the brain of transgenic mice of different ages used as a model of SAD was accompanied by increased Aβ levels and memory dysfunction, as measured by the MWM test. On the other hand, inhibiting HDAC3 prevented this dysfunction and decreased Aβ levels (Zhu et al. 2017). Guided by their research findings, Bardai and D’Mello (2011) suggested that the toxic effect of HDAC3 was specific to neuronal cells and that GSK3β was able to phosphorylate HDAC3 directly. This led to the suggestion that the neurotoxic effect of active GSK3β could be facilitated through the activation of HDAC3 (Bardai and D’Mello 2011).

On the other hand, Singh (2021) found that VPA inhibited GSK3β-mediated cleavage of APP, lessened Aβ production, decreased neuritic plaque formation, and improved the memory deficits in AD transgenic mice (D. Singh et al. 2021). According to Wood et al. (2004), this process could have started upstream of the insulin signaling as cells receiving VPA were highly sensitized to insulin (Wood et al. 2005). VPA was also found by Zacharias et al. (2011) to activate IRS-1, phosphorylating and activating Akt (Zacharias et al. 2011), which according to Datta et al. (2014) helps in insulin signal transduction and cell proliferation (Datta et al. 1999).

Authors of several studies used VPA and noted HDAC and GSK3 inhibition with an increase in histone acetylation (Brandt et al. 2006; Dash et al. 2010; Li et al. 2008; Miller et al. 2009). VPA treatment of transgenic mice has also been shown to significantly increase p-GSK3β (Ser9) levels (Long et al. 2015; Xuan et al. 2015). Likewise, Xuan et al. (2015) reported that VPA treatment caused a reduction in both forms of Aβ (40 and 42) in multiple brain regions of transgenic mice (Xuan et al. 2015). Supporting the hypothesis that VPA effect on Aβ inhibition was greatly due to its ability to inhibit GSK3, Sofola et al. (2010) established that GSK3 inhibition decreased Aβ production in an adult fly experimental model (Sofola et al. 2010). In addition, in VPA-treated AD transgenic mouse model, Tau hyperphosphorylation at Ser396 and Ser262 was significantly reduced in the study conducted by Long et al. (2015) (Long et al. 2015). Moreover, it was noted that VPA-treated neural stem cells showed greater expression of β-catenin compared to untreated control cells (L. Wang et al. 2015).

Since VPA is approved by the FDA for treating several disorders, it is currently the subject of several clinical trials. According to the findings yielded by one phase 1 trial, therapeutic doses of VPA were well tolerated by several patients with possible SAD (Profenno et al. 2005). Likewise, it was posited that VPA may be beneficial in improving the behavior of some SAD patients (C. Dolder and Mckinsey 2010). A meta-analysis of the results obtained in extant clinical trials revealed that VPA administration to SAD patients led to a relief in psychopathological symptoms with a possible occurrence of neuro-protection (Tariot et al. 2002). Following their meta-analysis, Zhang et al. (2022) concluded that VPA may be a potential drug to aid in the treatment of dementia patients (C. Q. Zhang et al. 2022). Schifitto et al. (2006) performed a pilot study on HIV-infected patients, some of whom suffered from cognitive impairment. VPA was given twice daily and was found to be completely safe, while improving neuropsychological performance in the patients who suffered from cognitive impairment (Schifitto et al. 2006). Several authors also advised that it is optimal to use VPA in combination with other psychotropic medications in treating agitation or motor symptoms associated with dementia (C. R. Dolder et al. 2012; Haeckert et al. 2021). A recent finding reported by Zeng et al. (2019) suggests that treating transgenic mice with VPA resulted in an increased density of newly formed neurons and improved neuronal proliferation in the hippocampus, along with a marked improvement in learning and memory (Zeng et al. 2019). In another recent study, Candon et al. (2023) proposed that VPA prescribing for SAD patients has become more prevalent in recent years, suggesting that VPA can be repurposed for dementia patients (Candon et al. 2023).

## Data Availability

The authors confirm that the data supporting the findings of this study are available within the article and/or its supplementary materials.

## Abbreviations

Aβ: Amyloid beta
CNS: Central Nervous System
GSK3: Glycogen synthase kinase-3
ICV: Intracerebroventricular
NFTs: Neuro-fibrillary tangles
p-IRS-1: Phosphorylated insulin receptor substrate-1
SAD: Sporadic Alzheimer’s disease
STZ: Streptozotocin
VPA: Valproic acid
